# OBJECTIVE AND SUBJECTIVE POVERTY AMONG OLDER PEOPLE AND ITS ASSOCIATION WITH HEALTH, FUNCTION, AND MORTALITY

**DOI:** 10.1093/geroni/igac059.2426

**Published:** 2022-12-20

**Authors:** Jeremy Jacobs, Jochanan Stessman

**Affiliations:** Hadassah Medical Center, Mount Scopus, Jerusalem, Yerushalayim, Israel; Hadassah Medical Center, Jerusalem, Yerushalayim, Israel

## Abstract

We examined objective and subjective poverty between ages 70-95, comparing and contrasting frequency, associated subsequent medical, functional, psychosocial impairment, and mortality. Data collection by the Jerusalem Longitudinal Study, following a representative community dwelling sample (born 1920-1921), assessed at ages 70, 78, 85, 90, and 95. We defined "objective poverty" as sole income from "national old-age pension" necessitating "supplemental income benefit" from the National Institute for Social Security; "subjective poverty" as "financial difficulty getting through each month". Subjective and objective poverty differed: frequency of objective poverty remained 13% throughout follow-up period, while subjective poverty was more frequent at younger age, decreasing from 24% at ages 70-85, to 14% at ages 90-95. Subjective poverty was significantly associated with depression at all ages (from HR=3.2, CI 95%:1.73-5.93 at age 70, to HR=2.45, CI 95%:1.32-4.54 at age 95), and poor self-rated health (from HR=2.69, CI 95%:1.63-4.46 at age 70, to HR=1.96, CI 95%:1.11-3.43 at age 95), however no consistent associations with objective poverty was observed. Both objective and subjective poverty were associated with low levels of physical activity, impaired functional and cognitive status. Mortality was significantly increased among subjects with subjective poverty at ages 70, 85, and 90 (ranging from HR=1.33, CI 95%: 1.02-1.74 to HR=1.50, CI 95%:1.11-2.03), while objective poverty was significantly associated with increased mortality at ages 78 and 85 (HR=1.43, CI 95%:1.01-2.03, and HR=1.55, CI 95%:1.16-2.07 respectively). In conclusion, subjective and objective poverty differ in their association with health, functional and psychosocial status, and mortality between ages 70-95.

